# Processing, Mechanical and Optical Properties of Additive-Free ZrC Ceramics Prepared by Spark Plasma Sintering

**DOI:** 10.3390/ma9060489

**Published:** 2016-06-18

**Authors:** Clara Musa, Roberta Licheri, Roberto Orrù, Giacomo Cao, Diletta Sciti, Laura Silvestroni, Luca Zoli, Andrea Balbo, Luca Mercatelli, Marco Meucci, Elisa Sani

**Affiliations:** 1 Dipartimento di Ingegneria Meccanica, Chimica e dei Materiali, Unità di Ricerca del Consorzio Interuniversitario Nazionale per la Scienza e Tecnologia dei Materiali (INSTM)—Università degli Studi di Cagliari, via Marengo 2, Cagliari 09123, Italy; claramusa80@gmail.com (C.M.); roberta.licheri@dimcm.unica.it (R.L.); giacomo.cao@dimcm.unica.it (G.C.); 2 ISTEC-CNR, Institute of Science and Technology for Ceramics, Via Granarolo 64, Faenza 48018, Italy; diletta.sciti@istec.cnr.it (D.S.); laura.silvestroni@istec.cnr.it (L.S.); luca.zoli@istec.cnr.it (L.Z.); andrea.balbo@unife.it (A.B.); 3 Corrosion and Metallurgy Study Centre “Aldo Daccò”, Engineering Department, University of Ferrara, G. Saragat 4a, Ferrara 44122, Italy; 4 INO-CNR, National Institute of Optics, Largo E. Fermi, 6, Firenze 50125, Italy; luca.mercatelli@ino.it (L.M.); marco.meucci@ino.it (M.M.); elisa.sani@ino.it (E.S.)

**Keywords:** Spark Plasma Sintering, self-propagating high-temperature synthesis, ultra-high-temperature-ceramics, carbides, mechanical properties, optical properties

## Abstract

In the present study, nearly fully dense monolithic ZrC samples are produced and broadly characterized from microstructural, mechanical and optical points of view. Specifically, 98% dense products are obtained by Spark Plasma Sintering (SPS) after 20 min dwell time at 1850 °C starting from powders preliminarily prepared by Self-propagating High-temperature Synthesis (SHS) followed by 20 min ball milling. A prolonged mechanical treatment up to 2 h of SHS powders does not lead to appreciable benefits. Vickers hardness of the resulting samples (17.5 ± 0.4 GPa) is reasonably good for monolithic ceramics, but the mechanical strength (about 250 MPa up to 1000 °C) could be further improved by suitable optimization of the starting powder characteristics. The very smoothly polished ZrC specimen subjected to optical measurements displays high absorption in the visible-near infrared region and low thermal emittance at longer wavelengths. Moreover, the sample exhibits goodspectral selectivity (2.1–2.4) in the 1000–1400 K temperature range. These preliminary results suggest that ZrC ceramics produced through the two-step SHS/SPS processing route can be considered as attractive reference materials for the development of innovative solar energy absorbers.

## 1. Introduction

Due to the peculiar combination of its chemico-physical and mechanical properties, such as high melting temperature (above 3500 °C), hardness, low density, chemical inertness, good electrical and thermal conductivity, zirconium carbide (ZrC) has been acknowledged as a very promising material for high temperature applications [1]. Moreover, ZrC displays low neutron absorption and selective solar energy absorption, which makes it particularly attractive in the nuclear [2] and solar energy [3] fields, respectively.

Various synthesis routes are currently available for the preparation of ZrC powders [4,5,6,7,8,9,10,11,12]. These include the carbo-thermal reduction of zirconia in high temperature furnaces [4], mechano-chemistry [5], solution methods [6], sol-gel [8], and self-propagating high-temperature synthesis (SHS) [7,9,10,11,12].

In spite of the availability of the various synthesis options reported above, the strong covalent Zr-C bond makes the fabrication of dense monolithic ZrC bodies a difficult achievement. For instance, it was reported that the pressureless sintering of pure ZrC powders for 60 min at 1950 °C leads to extremely porous samples (about 70% relative density) [13]. Final density was increased up to 94.4% only upon sintering at 2100 °C for 2 h [14]. The densification behavior is generally improved in the presence of mechanical loads, although the theoretical density is hard to be reached when considering classical hot pressing methods [15,16]. For example, 91% of dense samples are produced after 1 h of holding time at 2000 °C and 30 MPa applied pressure [16]. Modest densification levels are also reached when the reaction synthesis and densification of pure ZrC was performed in one single processing stage by reactive hot-pressing [14]. In contrast, significant improvements can be obtained when considering the Spark Plasma Sintering (SPS) technology, an efficient consolidation method where powder compacts are rapidly heated by the electric current flowing through the conductive die containing them [17]. For this reason, various studies involving the use of the SPS method for the densification of ZrC powders were carried out in the last decade [18,19,20,21,22,23]. Specifically, bulk products with relative density above 97% are generally obtained from as-received commercial ZrC powder by SPS when operating at temperatures of 2100 °C or higher values [18,20,23]. A mechanical treatment of the starting powders was reported to promote their densification by SPS [20,22]. Moreover, about 97.9% dense ZrC samples were recently obtained by SPS at 1800 °C, when the applied pressure was increased to 200 MPa, which was made possible by the use of a specifically designed double-die configuration [23].

Likewise for other members of ultra-high temperature ceramics (UHTCs), the consolidation of ZrC powders can be also made easier by the introduction of appropriate sintering aids, although the presence of secondary phases might not be desirable for certain high-temperature applications.

The difficulties encountered for the fabrication of fully dense ZrC products are also responsible for the lack of reliable data for related key properties, particularly for the thermo-mechanical and optical ones, which, on the other hand, are necessary to define the possible exploitation of the material in high-temperature solar absorbers.

This study deals with the fabrication of dense monophasic zirconium carbide by combining the SHS and SPS techniques. Specifically, according to previous findings providing evidence of the improved sintering ability of combustion synthesized powders with respect to differently prepared products [24,25], the zirconium carbide phase is first obtained by SHS. A systematic investigation is then performed to identify the optimal SPS temperature and time conditions to obtain high densification levels. The resulting bulk products are finally characterized from the microstructural, thermo-mechanical and optical points of view.

## 2. Materials and Methods

Zirconium (Alfa Aesar, product code 00418, particle size < 44 µm, purity > 98.5%, Karlsruhe, Germany) and graphite (Sigma-Aldrich, product code 282863, particle size < 20 µm, purity > 99.99%, St. Louis, MO, USA) powders were used as starting reactants for the synthesis of ZrC by SHS. Mixing of reagents was performed in agreement with the following reaction stoichiometry:

Zr + C → ZrC
(1)

About 15 g of the elemental powders were mixed for 20 min by means of a SPEX 8000 shaker mill (SPEX CertiPrep, Metuchen, NJ, USA) using plastic vials and 6 zirconia balls (2 mm diameter, 0.35 g) (Union process, Akron, OH, USA). SHS experiments were then conducted on cylindrical pellets (10 mm diameter, 20–30 mm height) prepared by uni-axially pressing 8–10 g of the obtained mixture. The synthesis process was carried out inside a reaction chamber under Ar atmosphere. An electrically heated tungsten wire (Inland Europe, Varigney, France) was used to locally activate the SHS reaction. The combustion temperature during synthesis evolution was measured using a two-color pyrometer (Ircon Mirage OR 15-990, Santa Cruz, CA, USA) focused on the center of the lateral surface of reacting samples. Combustion front velocity was estimated on the basis of the frame-by-frame analysis of the video recording. Further details relative to SHS experiments can be found elsewhere [26].

The SHS products were converted to powder form by subsequent ball milling (SPEX CertiPrep, Metuchen, NJ, USA). Specifically, about 4 g of the obtained porous samples have been mechanically treated using a stainless steel vial (VWR International PBI, Milan, Italy) with two steel balls (13 mm diameter, 4 g) to obtain a ball-to-powder weight, or charge ratio (CR), equal to two. The milling time (*t_M_*) was varied in the range 5–120 min. Particle size of the resulting powders was determined using a laser light scattering analyzer (CILAS 1180, Orleans, France).

Iron contamination from milling media to ZrC powders was evaluated by means of Inductively Coupled Plasma Optical Emission Spectroscopy (ICP-OES, Optima 5300DV Perkin Elmer, Waltham, MA, USA). The complete powder dissolution for this analysis was obtained using a hot mixture of nitric acid and hydrochloric acid (Carlo Erba Reagents, Milan, Italy) in a molar ratio of 1:3 (*aqua regia*).

An SPS equipment (515 model, Sumitomo Coal Mining Co., Ltd., Kanagawa, Japan) was used under vacuum (20 Pa) conditions for consolidation of the differently ball-milled SHS powders. This apparatus basically consists of a DC pulsed current generator (10 V, 1500 A, 300 Hz) (Sumitomo Coal Mining Co., Ltd., Kanagawa, Japan) combined with a uniaxial press (max 50 kN). The pulse cycle of the SPS machine was set to 12 ms on and 2 ms off, with a characteristic time of each pulse equal to 3.3 ms. About 3.6 g of powders were first cold-compacted inside the die (30 mm outside diameter; 15 mm inside diameter; 30 mm height) to produce 14.7 mm diameter specimens. To facilitate sample release after sintering, the internal die surface was previously lined with graphite foils (0.13 mm thick, Alfa Aesar, Karlsruhe, Germany). In order to control the evolution of the SPS process, temperature, current intensity, voltage, and sample displacement were monitored and recorded in real time. In particular, temperature was measured using a C-type thermocouple (W-Re, 250 µm diameter, Fuji Electronic Industrial Co., Ltd., Kanagawa, Japan) inserted in a small hole drilled on the die. SPS experiments were initiated with the imposition of a prescribed thermal cycle, where the temperature was first increased from the room value to the maximum level (*T_D_*) in 10 min (*t_H_*). Then, the *T_D_* value was kept constant for a prescribed duration (*t_D_*). The effect of *T_D_* and *t_D_* on the density of the sintered product was investigated in the 1750–1850 °C and 5–20 min ranges, respectively. The mechanical pressure of 50 MPa was applied from the beginning of each SPS experiment. For the sake of reproducibility, each experiment was repeated at least twice. Additional detailed information on SPS experiments is reported in previous studies [27,28].

In addition to the standard specimens (14.7 mm diameter), larger size samples were prepared for the mechanical characterization, in particular for the evaluation of flexural strength properties. However, significantly higher current levels with respect to those ones obtainable with the SPS 515 equipment (Sumitomo Coal Mining Co., Ltd., Kanagawa, Japan) (1500 A at most) are required to densify such larger samples. Thus, another SPS apparatus, namely the HPD 25-1 model (FCT Systeme GmbH, Rauenstein, Germany), able to provide current intensities up to 8000 A, was used to produce 40 mm diameter samples. In this regard, it should be noted that, for the latter equipment, temperature is measured and controlled at an axial point, whereas, as specified above, in the Sumitomo apparatus, such measurements are made on the surface of the die.

Relative densities of the sintered specimens were determined by the Archimedes’ method using distilled water as immersion medium and by considering the theoretical value for ZrC equal to 6.73 g/cm^3^ [29].

The crystalline phases were identified using an X-ray diffractometer (Philips PW 1830, Almelo, The Netherlands) equipped with a Ni filtered Cu K_α_ radiation (*λ* = 1.5405 Å). A Rietveld analytical procedure was employed to evaluate the average crystallite size [30]. The morphology of SHSed powders, as well as the microstructure and local phase composition of the sintered samples, were examined by scanning electron microscopy (SEM) (mod. S4000, Hitachi, Tokyo, Japan and mod. ΣIGMA, ZEISS NTS Gmbh, Oberkochen, Germany) and energy dispersive X-rays spectroscopy (EDS) (Kevex Sigma 32, Noran Instruments, Middleton, WI, USA and mod. INCA Energy 300, Oxford Instruments, Abingdon, UK), respectively.

Vickers microhardness (HV1.0) was measured with a load of 9.81 N, using a Zwick 3212 tester (Zwick GmbH & Co., Ulm, Germany), according to the European standard prEN 843-4.

With the same Zwick 3212 tester, the fracture toughness (K_IC_) was evaluated by the direct crack measurement method on the polished surfaces with a load of 98.1 N, by considering a radial-median crack and using the equation of Evans and Charles [31].

The 4-pt flexural strength (*σ*) was measured at room temperature (RT) and up to 1200 °C in partially protective Ar atmosphere, using the guidelines of the European standards for advanced ceramics ENV843-1:2004 [32] and EN820-1:2002 [33], respectively. Chamfered type-A bars with dimensions 25.0 × 2.5 × 2.0 mm^3^ (length by width by thickness, respectively) were tested at RT using a semi-articulated steel-made 4-pt fixture (lower span 20 mm, upper span 10 mm) in a screw-driven load frame (Zwick-Roell mod. Z050, Ulm, Germany), 1 mm/min of cross-head speed. Flexural strength at a high temperature was instead measured in a partially protective Ar environment using an adapted furnace (mod. HTTF, Severn Furnaces Ltd., Draycott Business Park, Durseley, UK) mounted on an Instron apparatus (mod. 6025) (Instron, Illinois Tool Works Inc., Norwood, MA, USA) using a 4-pt fixture made of Al_2_O_3_. Before applying the load during testing at high temperature, a dwell time of 18 min was set to reach thermal equilibrium. For each temperature, at least 3 bars were tested.

The surface topological characterization of the samples used for optical measurement was carried out with a non-contact 3D profilometer (Taylor-Hobson CCI MP, Leicester, UK), equipped with a 20X magnification objective lens. Two distinct areas of 0.08 × 1 cm^2^, were scanned at the center of the samples along two orthogonal directions and the topography data were analysed with the software Talymap 6.2 (Taylor-Hobson, Leicester, UK). The evaluation of 2D texture parameters was performed on 6 different profiles (3 for each area) extracted from the 3D data. The cut-off (*λc*, gaussian filter) applied for the separation of the roughness and waviness components was set according to the ISO 4288:2000 [34]. The 2D parameters were calculated as an average of the estimated values on all sampling lengths over each profile.

The 3D parameters were evaluated on the two scanned areas after removal of the surface form, short-wave and long-wave surface components (S-L surfaces) by applying a fifth order polynomial, 2.5 µm S-filter and 0.08 mm L-filter nesting indexes, respectively.

Hemispherical reflectance spectra were acquired using a double-beam spectrophotometer (Lambda900 by Perkin Elmer, Waltham, MA, USA) equipped with a Spectralon^®^-coated integration sphere (Perkin Elmer, Waltham, MA, USA) for the 0.25–2.5 µm wavelength region and a Fourier Transform spectrophotometer (FT-IR “Excalibur” by Bio-Rad, Hercules, CA, USA) equipped with a gold-coated integrating sphere and a liquid nitrogen-cooled detector for the range 2.5–16.5 μm.

## 3. Results and Discussion

### 3.1. Powder Synthesis and Consolidation

Neither compositional nor crystallite size changes were observed after mixing the elemental powders before the SHS stage. This feature is strictly ascribed to the extremely mild milling conditions correspondingly adopted, *i.e.*, CR = 0.14, and *t_M_* = 20 min. For the sake of comparison, it should be noted that when Zr and graphite powders were mechanically treated using a CR value of about 5.7 [5], line reflections of carbon disappeared from the XRD patterns after 2 h of milling, and the occurrence of an exothermic reaction was evident 20 min later. Such conditions are much more severe with respect to those applied in the present work, where milling of reactants was only aimed at obtaining a homogeneous mixture to be processed by SHS.

The favorable formation enthalpy of reaction (1), *i.e.*, ($\Delta H_{f}^{0}$ = −196.648 kJ/mol) [35], makes this reacting system prone to evolve under the combustion regime. In this regard, it should be noted that the synthesis and the simultaneous consolidation of ZrC from its elements might be not be convenient, as recently pointed out for similarly behaving systems processed by reactive SPS [28]. Indeed, when the reaction for the synthesis of ZrB_2_ was allowed to occur under the combustion regime, the impurities initially present in the raw powders gave rise to the sudden formation of gases, which could only barely escape from the confined graphite crucible. As a result, non-homogeneous microstructures and residual porosities were present in the obtained bulk products. Moreover, additional drawbacks (die/plungers breakage, safety problems, *etc.*) arose during the process. Based on such considerations, the synthesis by SHS of zirconium carbide and the densification of the resulting powders were carried out in two distinct steps in this work.

The high exothermic character of reaction (1) was confirmed by the self-propagating behavior exhibited by the reaction system as the pellet was locally ignited. Specifically, the generated combustion front spontaneously travelled through the specimen with average velocity and combustion temperature values equal to 8.5 ± 0.1 mm/s and 2390 ± 80 °C, respectively. The latter values are relatively lower with respect to those ones reported in previous studies related to the synthesis of ZrC by SHS from elemental reactants [9,10,11]. For instance, front velocity and combustion temperature of 1 cm/s and 2900 °C, respectively, were obtained by Lee *et al.* [9] in presence of 15 wt % ZrC as diluent. Moreover, combustion temperature values of up to 3100 °C were recorded by Song *et al.* [10] during the synthesis process. Such discrepancies can be mainly ascribed to the use of carbon black instead of graphite as starting reactant. Indeed, relatively higher reactivity, with respect to the synthesis carried out using graphite, is clearly obtained in the first case because of the amorphous nature and the finer particle sizes (down to the nano-scale) of C black. In addition, the high-energy ball milling treatment received by the initial mixture in Song *et al.* [10] investigation further promoted powder reactivity.

**Figure 1:**
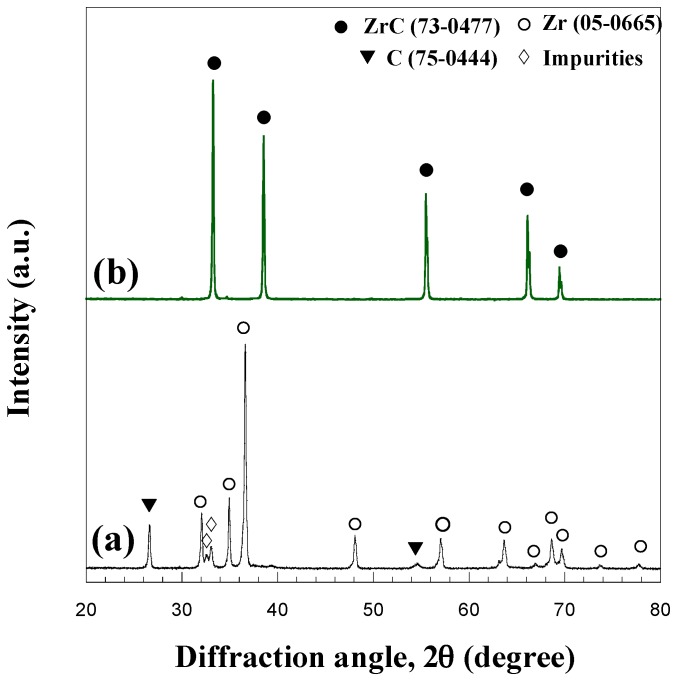
XRD patterns of starting reactants (**a**); and SHS product (**b**).

The XRD analysis of the synthesis product shown in Figure 1 indicates that, during SHS, the original reactants are completely converted with the exclusive formation of the desired cubic ZrC phase. It should be also noted that, in accordance with the typical self-cleaning character displayed by SHS processes [36], the small amount of unidentified impurities initially present in the starting powders were not detected in XRD spectra of the synthesis product.

The powders obtained after ball milling the SHS product for different time intervals have been characterized in terms of particles size and morphology. The results related to the granulometry measured by laser light scattering analysis are summarized in Table 1. As expected, all of the particles size parameters decreased as the milling time was increased from 5 to 20 min. In particular, the average diameter (*d_av_*) was correspondingly reduced from about 12.5 to less than 5 μm. In contrast, a peculiar behavior was observed when the *t_M_* value was further prolonged to 2 h. Indeed, while the *d*_10_ and *d*_50_ values still decreased, to indicate that the smaller particles were monotonically reduced during milling, the *d*_90_ parameter increased with respect to that measured after 20 min milling time. Correspondingly, the resulting average diameter value (about 9 μm) was also relatively higher as compared to the measurement obtained at the previous time point.

An increase in particle size, as revealed by laser-scattering analysis, was also recently observed during ball milling of commercial ZrC powders [20]. In particular, particle agglomeration was displayed when, after sufficiently long time milling treatments, the crystallite size was reduced at the nanoscale. Such outcome was attributed to cold-welding phenomena provoked by impacts taking place between milling media and powders. The occurrence of cold-welding is typically observed during milling of metal powders, as a consequence of their plastic deformation. On the other hand, it is less likely that such a mechanism has a major role when hard refractory ceramics like ZrC, which exhibit a fragile behavior, are mechanically processed, at least under the experimental conditions adopted in the present work.

To better clarify this issue, the milled powders were also examined by SEM, as reported later.

Iron contamination of milled powders from vials and balls, both made of steel, was also determined. The obtained values were less than 0.06 wt % and 0.12 wt % for the cases of ZrC powders milled for 20 min and 120 min, respectively. These values are both rather low, albeit iron contamination tends to become significant when the mechanical treatment is carried out for 2 h or longer times.

**Figure 2:**
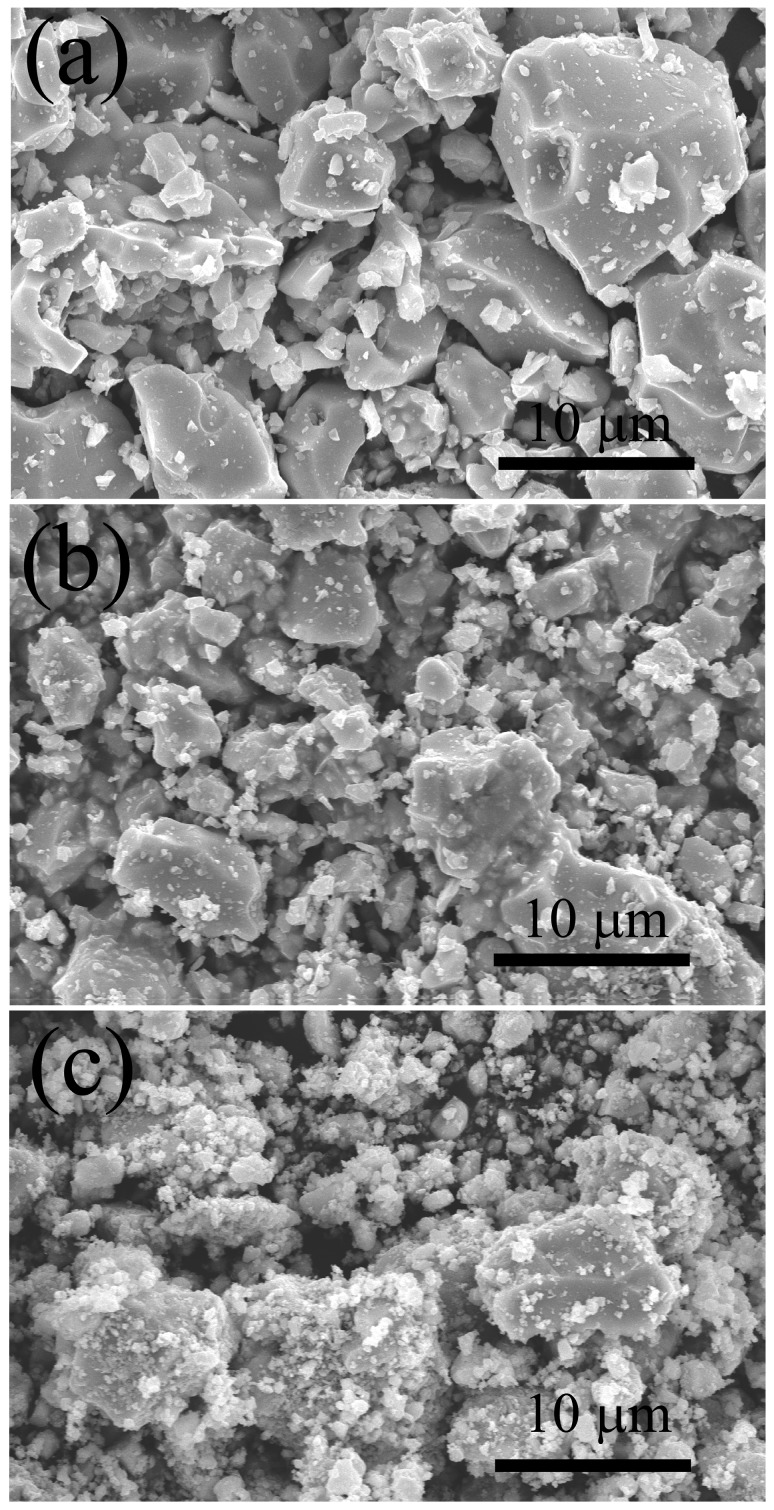
SEM images of SHS ZrC powders milled at different time intervals (*t_M_*): 5 min (**a**); 20 min (**b**); and 120 min (**c**).

The SEM images reported in Figure 2a indicate that powders processed for 5 min mainly consist of large particles whose size is even larger than 10 µm, while a minor amount of few micrometer sized grains is detected. Particle refinement below 10 µm clearly took place when the milling time was increased from 5 to 20 min (*cfr.* Figure 2b). These outcomes are perfectly consistent with the results obtained by laser scattering analysis, as reported in Table 1. The SEM micrograph shown in Figure 2c indicates that when the milling treatment was prolonged to 2 h, the relative amount of smaller particles, whose size was further reduced, increased. In addition, this feature is in agreement with data reported in Table 1. On the other hand, some discrepancies are found when examining the relatively larger particles obtained for *t_M_* = 2h. Specifically, the maximum size of each individual particle, generally less than 10 µm, changed only modestly with respect to those ones resulting after 20 min milling. However, the difference is that, in the first case (*t_M_* = 2 h), particles are extensively covered by several submicron-sized grains. This feature explains why laser scattering analysis provided higher *d*_90_ and *d_av_* values (*cfr.* Table 1) with respect to 20 min milled powders, despite of the prolonged ultrasonic treatment carried out. Based on these observations, the formation of such aggregates is more probably due to electrostatic charging phenomena induced by the milling process, although the contribution of cold-welding phenomenon is not completely excluded.

Table 1 also shows the progressive crystallite refinement of ZrC powders during the milling treatment. Specifically, it is seen that the powders mechanically processed for 20 min and 2 h display average crystallite sizes of about 140 nm and 45 nm, respectively. On the other hand, the size of 252 nm resulting from the application of the Rietveld analytical procedure on the XRD pattern of 5 min milled powders was above the threshold limit (about 200 nm) for this method. The effect of milling treatment of commercial ZrC powders was also recently taken into account by Núñez-Gonzalez *et al.* [20]. The relatively smaller crystallite size, down to 20 nm, obtained in the latter study for similar milling time conditions can be ascribed not only to the differences in the starting powders (3 µm particles sized), but, mainly, to the significantly more intense mechanical treatment, as demonstrated by the charge ratio equal to four, which is twice with respect to the value adopted in the present work.

**Figure 3:**
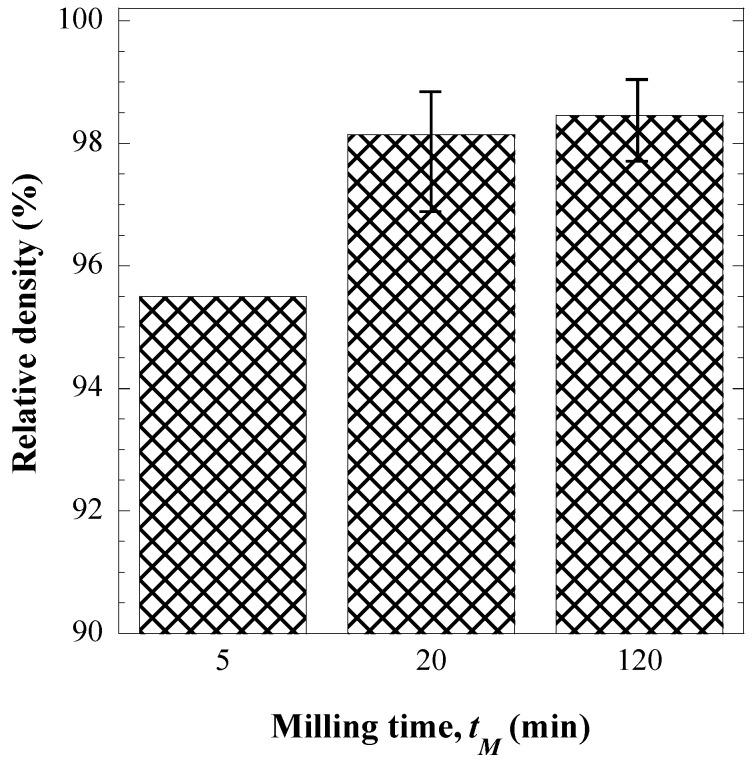
Effect of milling time on the relative density of ZrC samples obtained by SPS (*T_D_* = 1850 °C, *t_D_* = 10 min, *p* = 50 MPa).

The more convenient milling time (*t_M_*), in the range 5–120 min, for the SHS powders to be consolidated was then identified under prescribed SPS conditions, *i.e.*, *T_D_* = 1850 °C and *t_D_* = 20 min. The obtained results shown in Figure 3 indicate that an increase of the *t_M_* value from 5 to 20 min gives rise to a marked enhancement in powder densification. In contrast, only modest benefits are obtained when the powders milled for 2 h were consolidated by SPS. This outcome can be associated to the corresponding particle size evolution during the mechanical treatment (*cfr.* Figure 2 and Table 1), *i.e.*, the main changes are mostly confined to the milling time interval 5–20 min.

Along this line, it was recently reported that the SPS conditions needed to obtain nearly fully dense zirconium diboride and carbide products were significantly affected by the mechanical treatment previously received by the powders, only if crystallite size is reduced at the nanoscale [20,37]. In particular, the temperature level required to achieve, under 75 MPa mechanical pressure, the complete or near-complete densification of ZrC was lowered from 2100 °C (3 min holding time), for the case of as-received powders, to about 1850 °C (no socking time), if the crystallite size was decreased down to 20 nm through a high-energy ball milling treatment carried out up to 3 h with CR = 4 [20]. Such results are quite consistent to those ones obtained in the present work, where similar densification levels are also reached at 1850 °C when using 20 min or 120 min milled powders. The shorter sintering time adopted by Núñez-Gonzalez *et al.* [20] could be likely explained by the relatively finer crystallite size of the powders processed by SPS, as a consequence of the more severe milling treatment applied. It should be noted that higher milling intensities were not considered in the present work to avoid an excessive iron contamination from milling media, which would negatively affect the high-temperature properties of the resulting material. According to the consideration above, only the SHS powders milled for 20 min have been processed by SPS hereinafter, and the related results will be described and discussed in what follows.

**Figure 4:**
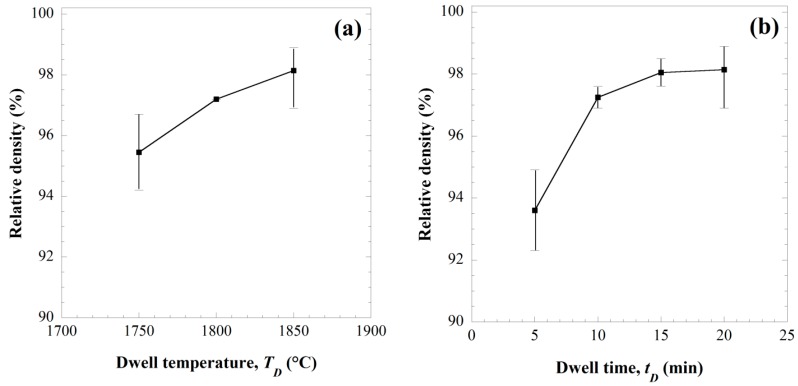
Effect of (**a**) the sintering temperature (*t_D_* = 20 min, *p* = 50 MPa, *t_H_* = 10 min) and (**b**) dwell time (*T_D_* = 1850 °C, *p* = 50 MPa, *t_H_* = 10 min) on the density of ZrC samples obtained by SPS.

Figure 4a shows the effect of the sintering temperature in the 1750–1850 °C range on the consolidation level when maintaining constant the holding time (*t*_D_ = 20 min), and applied pressure (50 MPa). As expected, higher dense samples are produced as the sintering temperature was progressively augmented. Along the same direction, the data plotted in Figure 4b indicate that an increase of the holding time from 5 to 20 min significantly promotes the consolidation of ZrC powders. Nonetheless, no additional beneficial effects are obtained when the processing time was further prolonged.

Thus, within the experimental conditions considered in the present work, the optimal *T_D_* and *t_D_* values able to guarantee the fabrication of about 98% (average value) dense ZrC products by SPS are 1850 °C and 20 min, respectively. It should be noted that such conditions are among the mildest one reported in the literature for the obtainment of similar density levels by SPS when using commercial ZrC powders [8,18,19,20,23]. The only exception is represented by the slightly lower temperatures (1800 °C) recently adopted by Wei *et al.* [23] to obtain 97.87% dense samples when taking advantage of a special double-die tooling setup, which enables the application of considerably higher mechanical pressures (200 MPa).

**Figure 5:**
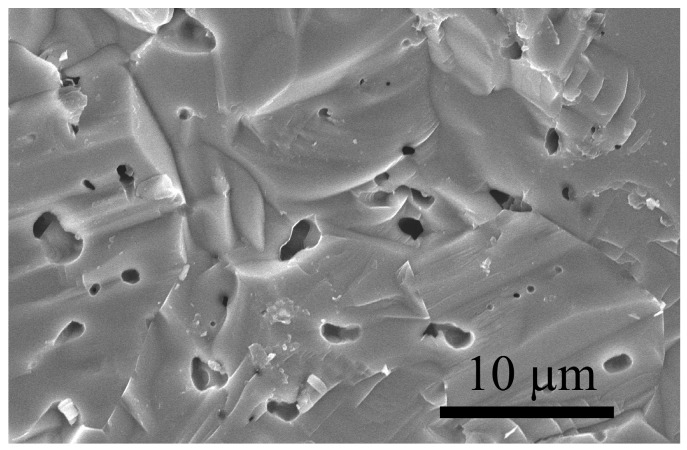
SEM image of the fracture surface of ZrC product obtained by SPS (*T_D_* = 1850 °C, *p* = 50 MPa, *t_H_* = 10 min, *t_D_* = 20 min).

A SEM micrograph showing the fracture surface of a dense ZrC sample obtained by SPS at *T_D_* = 1850 °C and *t_D_* = 20 min is reported in Figure 5. This image shows a transgranular fracture and a good level of consolidation, although a residual amount of closed porosity is present, with pore size in the range 1–3 µm.

**Figure 6:**
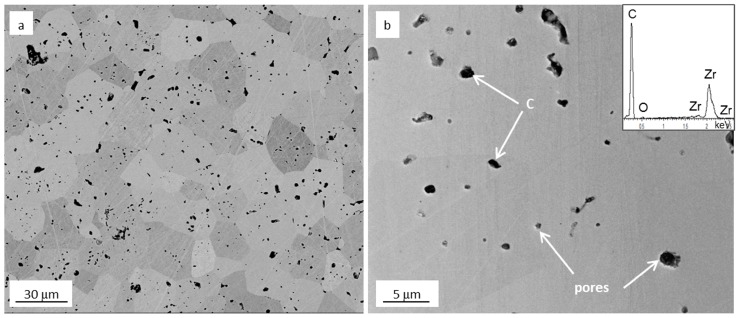
SEM images of the polished surface of the SHS-SPSed ZrC sample showing (**a**) a microstructure overview in back-scattered image mode and (**b**) the presence of closed porosity and carbon inclusions in In-lens mode with the EDS spectrum of C-rich pockets inset.

A backscattered image collected on the polished surface (Figure 6a) shows the presence of black spots. From the In-lens image (Figure 6b), it can be appreciated that the latter ones are either pores or residual carbon inclusions (see EDS spectrum). The total amount of carbon inclusion and pores is, however, below 7 vol %.The mean grain size of ZrC grains is around 15 ± 5 μm, with minimum grains size around 8 μm and maximum value around 28 μm, *i.e.*, higher than monolithic ZrC obtained through SPS of commercial ZrC powders, which resulted to be about 13 μm [18]. It is presumed that the ultrafine powder fraction coalesced on the coarser particles to generate larger final grains when exposed to the high temperature conditions established during the sintering process.

As reported in the Section 2, the 40 mm diameter samples needed for the evaluation of thermo-mechanical properties, as reported in the next section, have been produced taking advantage of an SPS apparatus (FCT Systeme GmbH, Rauenstein, Germany), different from that one (Sumitomo, Kanagawa, Japan) considered so far to obtain 14.7 mm sized specimens, the latter one being unable to provide the required higher electric current levels. In this regard, it should be noted that, although the two pieces of equipment are based on the same principle, they display some differences in the temperature measurements. In particular, for the Sumitomo machine, the temperature is monitored and controlled at the surface of the die, while such measurement is made at an axial point for the FCT equipment. Thus, to compensate the thermal gradients present along the radial direction, the dwell temperature value set in the latter case was increased from 1850 °C to 1950–2000 °C, while the holding time and the applied pressure remained unchanged, *i.e.*, 50 MPa and 20 min, respectively. Correspondingly, the obtained products are characterized by density of 97.3% or higher values, which fall within the error bar of the data reported in Figure 4 for smaller diameter samples. Moreover, SEM analysis performed on the resulting SPSed products indicated that the ZrC grains size were also comparable in the two cases. Based on these findings, it is possible to state that SHS powders undergoing sintering are subjected to similar thermal cycles in the two machines.

### 3.2. Mechanical Characterization

The hardness of monolithic ZrC samples obtained in this work by SPS was 17.5 ± 0.4 GPa. This value is in very good agreement with those previously found on monophasic ZrC produced through SPS from commercial powders, *i.e.*, 17.9 ± 0.6 GPa [18]. Higher values could be likely obtained if the mean grains size, residual porosity and carbon inclusions are significantly reduced.

As for fracture toughness, the resulting mean K_IC_ value, 2.6 ± 0.2 MPa·m^0.5^, is slightly larger than the one found for ZrC-based materials containing 1 vol % MoSi_2_, *i.e.*, 2.1 ± 0.2 MPa·m^0.5^ [18]. The brittleness of this material is also testified by the tendency of chipping of the surface during loading or immediately after the releasing of the load.

The room temperature strength of the ZrC products resulting from SHSed powders was 248 ± 66 MPa, in the same order of the values reported in the literature for ZrC-based ceramics produced by pressureless sintering [13]. Nonetheless, it should be noted that these values are significantly lower than those found for monolithic ZrC obtained by SPS from commercial powder (H.C. Starck, Grade B, Karlsruhe, Germany) [18], 407 ± 38 MPa, although, in the latter case, strength data refer to three-point bending strength performed on smaller bars (1.0 mm × 0.8 mm × 10 mm). Such discrepancies between monolithic samples obtained from SHS or commercial powders could be due to the different grain size distribution of the two materials, as mentioned above, and to the presence of different volume amount of free carbon, 5 vol % and 2 vol %, respectively. However, it should be noted, that the bending strength of the ceramic produced by SHS/SPS remained basically unaltered, *i.e.*, 247 ± 36 MPa, when the test was carried out at 1000 °C. Only when the testing temperature was further increased to 1200 °C was a notable drop down to 85 MPa observed. Correspondingly, the monolithic sample resulted as being strongly exfoliated at such a temperature, probably owing to residual traces of oxygen remained trapped in the furnace during the test.

### 3.3. Optical Properties

Before performing the optical measurements, the polished sample surface was preliminarily characterized from the topological point of view. The mean values of the 2D and 3D surface texture parameters, obtained according to the ISO 4287:1997 [38] and ISO 25178-2:2012 [39], respectively, are reported in Table 2 along with the corresponding standard deviations.

Ra and Rq represent the most commonly amplitude parameters used in surface texture characterization and provide the average roughness. The Rq parameter has a more statistical significance with respect to Ra and is related, as the 3D height parameter Sq, to the surface energy and to the amount of light scattered from smooth surfaces [40]. The analyzed surfaces show very low values of both Ra (0.009 µm) and Rq (0.014 µm).

However, Ra and Rq do not give information about the shape of surface irregularities and the abnormal peaks or valleys do not affect significantly their values. The skewness and kurtosis parameters are related to defects distribution of the studied surface: negative values of Rsk indicate the prevalence of pores or valleys, while Rku values higher than three indicate the presence of sharp defects. Specifically, the values of these parameters for the ZrC samples show that the distribution of the extracted profile heights is asymmetric, negative (Rsk = −0.91) and quite narrow, (Rku = 4.9). The parameter Rdq is related to the optical properties of the surface and low values, as for the present case (Rdq = 0.43), specify that the surface is a good reflector. The profile slope is very low. Therefore, the measured 2D amplitude parameters indicate that the studied surface is very smooth but is characterized by the presence of valleys or holes.

The values of the 3D parameters reported in Table 2 are higher than the corresponding 2D, probably because of the larger sampling area that may include a high number of surface defects. Particularly high is the value of the Sku parameter. This outcome may be due to the fact that this index is extremely sensitive to local defects (holes or valleys) and also to error propagation, since it depends on high order powers in its mathematical expression. In any case, the 3D parameters are in substantial agreement with the corresponding 2D parameters and confirm the previous findings.

**Figure 7:**
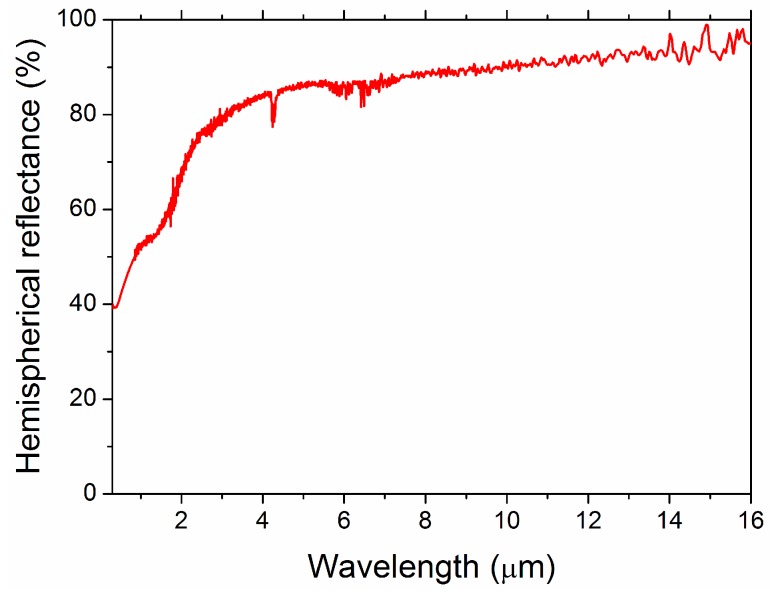
Hemispherical reflectance spectrum of ZrC sample.

Figure 7 shows the hemispherical reflectance of the ZrC specimen. It is possible to appreciate the typical step-like spectrum of UHTC carbides, characterized by a low reflectance (*i.e.*, a high optical absorption) in the visible-near infrared and a high reflectance plateau (*i.e.*, a low thermal emittance) at longer infrared wavelengths.

For solar absorber applications, sunlight absorption characteristics are quantified by the total directional solar absorbance at the temperature T:

(2) $${\alpha \prime }_{S}(T)=\frac{\int_{\lambda_{\min}}^{\lambda_{\max}}(1-{\rho \prime }^{\cap }(\lambda ,T))\times S(\lambda )d\lambda }{\int_{\lambda_{\min}}^{\lambda_{\max}}S(\lambda )d\lambda }$$

which is defined in terms of the spectral directional-hemispherical reflectance *ρ’^∩^*(*λ*,*T*) (*i.e.*, the hemispherical reflectance acquired for a given incidence direction of light, like it happens for measurements carried out with an integrating sphere) and of sunlight spectrum *S*(*λ*) [41]. The energy lost by thermal radiation by the absorber heated at the temperature T is connected to the total directional thermal emittance parameter expressed by:

(3) $$\varepsilon \prime_{\lambda 1,\lambda 2}(T)=\frac{\int_{\lambda 1}^{\lambda 2}(1-{\rho \prime }^{\cap }(\lambda ,T))\times B(\lambda ,T)d\lambda }{\int_{\lambda 1}^{\lambda 2}B(\lambda ,T)d\lambda }$$

where *B*(*λ*,*T*) is the blackbody spectrum at the temperature T. The *α*/*ε* ratio is called spectral selectivity and is connected to the material ability to respond differently to optical radiation in the sunlight and thermal emission spectral ranges. Thus, roughly speaking, an ideal solar absorber should have a high *α* ≈ 1, a low *ε* ≈ 0 and the highest as possible *α*/*ε* parameter. Under the remarks made by the authors in a recent work [42], the *ρ*’*^∩^*(*λ*,*T*) function can be approximated with the spectral hemispherical reflectance measured at room temperature.

For the sake of comparison of the sample tackled in the present work with analogous previously investigated systems, *i.e.*, sintering aid-doped ZrC [43] and fully dense monolithic ZrB_2_ obtained by SHS/SPS [44]. Parameters appearing in Equations (2) and (3) have been calculated by considering integration bounds *λ*_min_ = 0.3 μm, *λ*_max_ = 2.3 μm, *λ*_1_ = 0.3 μm, *λ*_2_ = 15.0 μm and temperatures of 1200 K and 1400 K. Correspondingly, *α* = 0.51, *ε*(1200 K) = 0.21, and *ε*(1400 K) = 0.24 are obtained for the case of the ZrC sample produced in this work, which leads to *α*/*ε*(1200 K) = 2.4, and *α*/*ε*(1400 K) = 2.1. Based on these results, it can be stated that the latter product is slightly less absorptive and emissive with respect to MoSi_2_-containing ZrC samples (*α* = 0.55–0.56 and *ε*(1200 K) = 0.23–0.27), while spectral selectivity (2.4–2.1) is similar [43]. In addition, when the comparison is extended to the bulk additive-free ZrB_2_ material recently processed following the same SHS/SPS route [40], it is found that the ZrC product appears slightly more absorptive and emissive, *i.e.*, *α*_ZrB2_ = 0.47, *ε*_ZrB2_(1400 K) = 0.18, with a slightly higher spectral selectivity displayed by the diboride system (*α*/*ε* = 2.6).

Finally, it should be mentioned that a possible criticism for UHTCs in solar applications is represented by the relatively low solar absorbance when comparing them, for instance, to silicon carbide. However, it has been recently demonstrated,in the case of hafnium carbide [45], that solar absorbance can be significantly increased by surface texturing without detrimentally affecting thermal emittance.

## 4. Conclusions

ZrC-based ultra-refractory ceramics are promising candidate materials for solar energy and application in other industrial fields. In spite of their technological interest, the conventional processing technologies generally utilized to obtain highly dense products require severe sintering conditions (temperatures above 2000 °C and dwelling times on the order of hours), unless sintering aids are introduced. In this work, about 98% dense monolithic ZrC samples are successfully produced by SPS after 20 min holding time at 1850 °C, being the starting powders preliminarily synthesized by SHS followed by 20 min ball milling treatment. The milder sintering conditions adopted in this work can be taken as an indication of the high sintering ability of the powder produced by SHS.

The measured Vickers hardness and fracture toughness of the resulting material, 17.5 ± 0.4 GPa and 2.6 ± 0.2 MPa·m^0.5^, respectively, are similar to the values reported in the literature for ZrC monoliths. The relatively low mechanical strength, about 250 MPa at room temperature, is likely associated to the coarse microstructure characterizing the bulk ceramic, with grain size up to 28 μm, as well as to the presence of residual pores and carbon inclusions. Nevertheless, the obtained room temperature strength remains basically unchanged up to 1000 °C, whereas such a property becomes significantly worse (85 MPa) as the temperature is further increased to 1200 °C. In this regard, the use of higher purity and finer initial powders are expected to improve the densification as well as the microstructure of the sintered product and, in turn, the resulting strength.

The topological 2D and 3D characterization of the polished sample exposed to optical measurements indicate that the studied surface is very smooth, albeit valleys or holes are also present.

Regarding the optical behavior, the obtained ZrC specimen displayed a step-like spectrum, typical of transition metal carbides, with high absorption in the visible-near infrared area and low thermal emittance at longer infrared wavelengths. Furthermore, the resulting spectral selectivity values are sufficiently good, 2.4 and 2.1 at 1000 K and 1400 K, respectively.

In summary, it is possible to conclude that the combination of the SHS and SPS routes provide highly dense ZrC products with performances for solar absorber applications comparable with those displayed by analogous UHTC materials, specifically monolithic ZrB_2_ obtained with the same synthesis/sintering techniques and additive-containing ZrC produced by alternative methods.

## Figures and Tables

**Table 1:** Particle size characteristics, as determined by laser scattering analysis, and crystallite size, obtained using the Rietveld analytical procedure, of differently ball-milled SHSed powders.

*t_M_* (min)	*d*_10_ (μm)	*d*_50_ (μm)	*d*_90_ (μm)	*d_av_* (μm)	*Crystallite Size* (nm)
5	1.50 ± 0.06	9.20 ± 0.11	29.90 ± 1.65	12.60 ± 0.25	>200
20	0.61 ± 0.03	3.68 ± 0.01	10.12 ± 0.21	4.59 ± 0.04	137.3 ± 1.2
120	0.11 ± 0.01	1.55 ± 0.32	34.10 ± 5.85	8.99 ± 1.58	44.2 ± 0.1

**Table 2:** Description and average (Av.) values of the measured two-dimensional (2D) and three-dimensional (3D) surface parameters.

2D Parameters	Av. Values	Description
**Ra (µm)**	0.009 ± 0.004	Arithmetic Mean Deviation of the roughness profile.
**Rq (µm)**	0.014 ± 0.006	Root-Mean-Square (RMS) Deviation of the roughness profile.
**Rsk**	−0.91 ± 0.15	Skewness of the roughness profile.
**Rku**	4.90 ± 0.56	Kurtosis of the roughness profile.
**Rp (µm)**	0.020 ± 0.012	Maximum Peak Height of the roughness profile.
**Rv (µm)**	0.047 ± 0.017	Maximum Valley Depth of the roughness profile.
**Rz (µm)**	0.068 ± 0.028	Maximum height of roughness profile on the sampling length.
**Rc (µm)**	0.043 ± 0.020	Mean height of the roughness profile elements.
**Rt (µm)**	1.29 ± 0.77	Total height of roughness profile (on the evaluation length).
**RSm (mm)**	0.076 ± 0.012	Mean Width of the roughness profile elements.
**Rdq**	0.43 ± 0.19	Root-Mean-Square Slope of the roughness profile.
**3D Parameters**	**Av. Values**	**Description**
**Sa** **(µm)**	0.070 ± 0.030	Arithmetic mean height of the S-L Surface.
**Sq** **(µm)**	0.56 ± 0.16	Root mean square height of the S-L Surface.
**Ssk**	−11.4 ± 5.2	Skewness of the S-L Surface.
**Sku**	150 ± 70	Kurtosis of the S-L Surface.
**Sp** **(µm)**	11.0 ± 3.10	Maximum peak height in the S-L Surface.
**Sv** **(µm)**	19.6 ± 1.60	Maximum pit height of the S-L Surface.
**Sz** **(µm)**	30.6 ± 4.7	Maximum height of the S-L Surface.

## References

[1] Upadhya K., Yang J.-M., Hoffman W.P. (1997). Materials for ultrahigh temperature structural applications. Am. Ceram. Soc. Bull..

[2] Katoh Y.G., Vasudevamurthy T., Nozawa L.L. (2013). Snead Properties of zirconium carbide for nuclear fuel applications. J. Nucl. Mater..

[3] Sani E., Mercatelli L., Francini F., Sans J.-L., Sciti D. (2011). Ultra-refractory ceramics for high-temperature solar absorbers. Scr. Mater..

[4] Maitre A., Lefort P. (1997). Solid state reaction of zirconia with carbon. Solid State Ion..

[5] Yen B.K. (1998). X-ray diffraction study of mechanochemical synthesis and formation mechanisms of zirconium carbide and zirconium silicides. J. Alloys Compd..

[6] Shen G., Chen D., Liu Y., Tang K., Qian Y. (2004). Synthesis of ZrC hollow nanospheres at low temperature. J. Cryst. Growth.

[7] Li J., Fu Z.Y., Wang W.M., Wang H., Lee S.H., Niihara K. (2010). Preparation of ZrC by self-propagating high-temperature synthesis. Ceram. Int..

[8] Xie J., Fu Z., Wang Y., Lee S.W., Niihara K. (2014). Synthesis of nanosized zirconium carbide powders by a combinational method of sol-gel and pulse current heating. J. Eur. Ceram. Soc..

[9] Lee H.B., Cho K., Lee J.W. (1995). Synthesis and temperature profile analysis of ZrC by SHS method. J. Korean Ceram. Soc..

[10] Song M.S., Huang B., Zhang M.X., Li J.G. (2009). In situ synthesis of ZrC particles and its formation mechanism by self-propagating reaction from Al–Zr–C elemental powders. Powder Technol..

[11] Zhang M.X., Hu Q.D., Huang B., Li J.G. (2011). Fabrication of ZrC particles and its formation mechanism by self-propagating high-temperature synthesis from Fe–Zr–C elemental powders. J. Alloys Compd..

[12] Song M., Ran M., Long Y. (2013). Synthesis of ultrafine zirconium carbide particles by SHS in an Al–Zr–C system: Microstructural evaluation and formation mode. J. Alloys Compd..

[13] Silvestroni L., Sciti D. (2008). Microstructure and properties of pressureless sintered ZrC-based materials. J. Mater. Res..

[14] Zhao L., Jia D., Duan X., Yang Z., Zhou Y. (2011). Pressureless sintering of ZrC-based ceramics by enhancing powder sinterability. Int. J. Refract. Met. Hard Mater..

[15] Nachiappan C., Rangaraj L., Divakar C., Jayaram V. (2010). Synthesis and Densification of monolithic zirconium carbide by reactive hot pressing. J. Am. Ceram. Soc..

[16] Wang X.-G., Liu J.-X., Kan Y.-M., Zhang G.-J. (2012). Effect of solid solution formation on densification of hot-pressed ZrC ceramics with MC (M = V, Nb and Ta) additions. J. Eur. Ceram. Soc..

[17] Orrù R., Licheri R., Locci A.M., Cincotti A., Cao G. (2009). Consolidation/synthesis of materials by electric current activated/assisted sintering. Mater. Sci. Eng. R.

[18] Sciti D., Guicciardi S., Nygren M. (2008). Spark Plasma Sintering and mechanical behavior of ZrC-based composites. Scr. Mater..

[19] Gendre M., Maitre A., Trolliard G. (2010). A study of the densification mechanism during spark plasma sintering of zirconium (oxy-) carbide powders. Acta Mater..

[20] Núñez-Gonzalez B., Ortiz A.L., Guiberteau F., Nygren M. (2012). Improvement of the Spark-Plasma-Sintering Kinetics of ZrC by High-Energy Ball-Milling. J. Am. Ceram. Soc..

[21] Sun S.-K., Zhang G.-J., Wu W.-W., Liu J.-X., Suzuki T., Sakka Y. (2013). Reactive spark plasma sintering of ZrC and HfC ceramics with fine microstructures. Scr. Mater..

[22] Bertagnoli D., Borrero-López O., Rodríguez-Rojas F., Guiberteau F., Ortiz A.L. (2015). Effect of processing conditions on the sliding-wear resistance of ZrC triboceramics fabricated by spark-plasma sintering. Ceram. Int..

[23] Wei X., Back C., Izhanov O., Khasanov O.L., Haines C.D., Olevsky E. (2015). Spark Plasma Sintering of Commercial Zirconium Carbide Powders: Densification Behaviour and Mechanical Properties. Materials.

[24] Mishra S.K., Das S., Pathak L.C. (2004). Defect structures in zirconium diboride powder prepared by self-propagating high-temperature synthesis. Mater. Sci. Eng. A.

[25] Licheri R., Orrù R., Musa C., Cao G. (2008). Combination of SHS and SPS Techniques for Fabrication of Fully Dense ZrB_2_-ZrC-SiC Composites. Mater. Lett..

[26] Cincotti A., Licheri R., Locci A.M., Orrù R., Cao G. (2003). A review on combustion synthesis of novel materials: Recent experimental and modeling results. J. Chem. Technol. Biotechnol..

[27] Musa C., Orrù R., Licheri R., Cao G. (2011). Spark Plasma Synthesis and Densification of TaB_2_ by Pulsed Electric Current Sintering. Mater. Lett..

[28] Licheri R., Musa C., Orrù R., Cao G. (2015). Influence of the heating rate on the in-situ synthesis and consolidation of ZrB_2_ by Reactive Spark Plasma Sintering. J. Eur. Ceram. Soc..

[29] Haynes W.M. (2012). CRC Handbook of Chemistry and Physics.

[30] Lutterotti L., Ceccato R., Dal Maschio R., Pagani E. (1998). Quantitative analysis of silicate glass in ceramic materials by the Rietveld method. Mater. Sci. Forum..

[31] Evans A.G., Charles E.A. (1976). Fracture toughness determination by indentation. J. Am. Ceram. Soc..

[32] (2004). Advanced Technical Ceramics—Monolithic Ceramics—Mechanical Properties at Room Temperature—Part 1: Determination of Flexural Strength.

[33] (2003). Advanced Technical Ceramics—Methods for Testing Monolithic Ceramics—Thermomechanical Properties—Part 1: Determination of Flexural Strength at Elevated Temperatures.

[34] (1998). Geometrical Product Specifications (GPS)—Surface Texture: Profile Method—Rules and Procedures for the Assessment of Surface Texture.

[35] Barin I. (1989). Thermochemical Data of Pure Substances.

[36] Varma A., Rogachev A.S., Mukasyan A.S., Hwang S. (1998). Combustion Synthesis of Advanced Materials: Principles and Applications. Adv. Chem. Eng..

[37] Zamora V., Ortiz A.L., Guiberteau F., Nygren M. (2012). Crystal-size dependence of the spark-plasma-sintering kinetics of ZrB2 ultra-high-temperature ceramics. J. Eur. Ceram. Soc..

[38] (1998). Geometrical Product Specifications (GPS)—Surface Texture: Profile Method—Terms, Definitions and Surface Texture Parameters.

[39] (2012). Geometrical Product Specifications (GPS)—Surface Texture: Areal—Part 2: Terms, Definitions and Surface Texture Parameters.

[40] Leach R.K. (2010). Fundamentals Principles of Engineering Nanometrology.

[41] (1989). Solar Spectral Irradiance.

[42] Sani E., Mercatelli L., Sansoni P., Silvestroni L., Sciti D. (2012). Spectrally selective ultra-high temperature ceramic absorbers for high-temperature solar plants. J. Renew. Sustain. Energy.

[43] Sani E., Mercatelli L., Meucci M., Balbo A., Silvestroni L., Sciti D. (2016). Compositional dependence of optical properties of zirconium, hafnium and tantalum carbides for solar absorber applications. Sol. Energy.

[44] Sani E., Mercatelli L., Meucci M., Balbo A., Musa C., Licheri R., Orrù R., Cao G. (2016). Optical properties of dense zirconium and tantalum diborides for solar thermal absorbers. Renew. Energy.

[45] Sciti D., Silvestroni L., Trucchi D.M., Cappelli E., Orlando S., Sani E. (2015). Femtosecond laser treatments to tailor the optical properties of hafnium carbide for solar applications. Sol. Energy Mater. Sol. Cells.

